# Intestinal colonization with *Campylobacter jejuni* affects broiler gut microbiota composition but is not inhibited by daily intake of *Lactiplantibacillus plantarum*

**DOI:** 10.3389/fmicb.2023.1205797

**Published:** 2023-07-28

**Authors:** Eliška Valečková, Li Sun, Helen Wang, Faruk Dube, Emma Ivarsson, Kamyar Mogodiniyai Kasmaei, Patrik Ellström, Helena Wall

**Affiliations:** ^1^Department of Animal Nutrition and Management, Swedish University of Agricultural Sciences, Uppsala, Sweden; ^2^Department of Medical Biochemistry and Microbiology, Uppsala University, Uppsala, Sweden; ^3^Department of Biomedical Science and Veterinary Public Health, Swedish University of Agricultural Sciences, Uppsala, Sweden; ^4^Department of Medical Sciences, Zoonosis Science Center, Uppsala University, Uppsala, Sweden

**Keywords:** *Lactobacillus plantarum*, *Campylobacter jejuni*, broiler, gut microbiota, qPCR, sequencing

## Abstract

**Introduction:**

Lactobacilli may prevent broilers from colonization with *Campylobacter* spp. and other gram-negative zoonotic bacteria through lactic acid production and modulation of the intestinal microbiota. This study evaluated the effects of daily intake of *Lactiplantibacillus plantarum* 256 (LP256) on *Campylobacter jejuni (C. jejuni)* loads in ceca and feces of *C. jejuni* challenged broilers, together with the changes in the gut microbiota.

**Methods:**

Two experiments were conducted using the broilers Ross 308 (R-308; Experiment 1) for 42 days and Rowan Ranger broilers (RR; Experiment 2) for 63 days. The *LP256* strain was administered either via silage inoculated with *LP256* or direct supplementation in the drinking water. Concurrently, haylage as a forage similar to silage but without any inoculum was tested. *C. jejuni* loads in fecal matter and cecal content were determined by plate counts and qPCR, respectively. The cecal microbiota, in response to treatments and the challenge, were assessed by 16S rRNA sequencing.

**Results and Discussion:**

Culturing results displayed a significant reduction in *C. jejuni* colonization (2.01 log) in the silage treatment in comparison to the control at 1 dpi (day post-infection) in Experiment 1. However, no treatment effect on *C. jejuni* was observed at the end of the experiment. In Experiment 2, no treatment effects on *C. jejuni* colonization were found to be statistically significant. Colonization load comparison at the peak of infection (3 dpi) to that at the end of the trial (32 dpi) revealed a significant reduction in *C. jejuni* in all groups, regardless of treatment. Colonization dynamics of *C. jejuni* in the cecal samples analyzed by qPCR showed no difference between any of the treatments in Experiment 1 or 2. In both experiments, no treatment effects on the cecal microbiota were observed. However, proportional changes in the bacterial composition were observed after the *C. jejuni* challenge, suggesting that colonization affected the gut microbiota. Overall, the daily intake of *LP256* was not effective in reducing *C. jejuni* colonization in either broiler type at the end of the rearing period and did not cause any significant changes in the birds’ cecal microbiota composition.

## 1. Introduction

The ceca are believed to have an important role in the gut health and performance of broiler birds. However, its role in the maintenance of gut health and modulation of the gut microbiota is still not fully understood. As the most densely colonized microbial habitat in broilers, its microbial density is estimated to be 10^11^–10^12^ bacterial cells per gram (Rinttilä and Apajalahti, 2013). The description and understanding of intestinal microbial communities and their interactions, are essential for the development of feed additives and dietary changes to improve broiler health, performance, and welfare (Sugiharto, 2016). A wide variety of feed supplements, such as prebiotics, probiotics, and organic acids, focus on the stabilization of the gut microbiota to secure intestinal health (Yang et al., 2009).

Probiotics are natural microbes that benefit their host fundamentally through their action in the gastrointestinal tract (Abd El-Hack et al., 2020). Single-strain probiotic species including, among others, species of *Bifidobacterium*, *Bacillus*, *Enterococcus*, *Streptococcus*, and *Lactobacillus* have previously shown positive effects on broiler performance, modulation of the gut microbiome as well as inhibition of pathogens through different principles, i.e., competitive exclusion, production of organic acids, or production of antimicrobial compounds (Neal-McKinney et al., 2012; Prabhurajeshwar and Chandrakanth, 2019; Krysiak et al., 2021). Furthermore, it has been shown that probiotics help to maintain microbial homeostasis thus avoiding colonization by pathogens, and may suppress *Campylobacter* colonization (Di Marcantonio et al., 2022).

Campylobacteriosis is the most commonly reported zoonosis in the European Union (EU), where broiler products are a common source of infection due to insufficient heat treatment or cross-contamination. According to the European Food Safety Authority (EFSA), 58% of human *Campylobacter jejuni* (*C. jejuni*) infections are associated with broiler meat (EFSA, 2020). Poultry feed with low pH and a high number of lactic acid bacteria (LAB) has been shown to reduce the susceptibility to *Campylobacter* colonization in broilers (Heres et al., 2003). This effect might be explained by the principles of pathogen inhibition mentioned above.

Although the prevalence of *Campylobacter* in conventional broiler production in Sweden is currently low, the problem remains in organic production. In 2021, 5% of tested conventional batches were *Campylobacter* positive at slaughter, whereas in organic production, 33% of tested flocks were positive (Swedish Poultry Meat Association, 2021). The higher frequency in the latter is due to the access to outdoor reservoirs of *Campylobacter*, as all organic poultry in the EU must have the opportunity to spend time outdoors (Commission Regulation (EC) 889/2008, 2008). In addition, organic poultry must be provided daily access to forage where silage is provided at some organic broiler farms (Crawley, 2015).

This study aimed to investigate the effects of daily intake of *Lactiplantibacillus plantarum* strain 256 (*L. plantarum* 256; *LP*256) on *C. jejuni* load in broiler’s cecum and feces, together with the changes in their gut microbiota. In organic farming, silage can be supplied as forage to the broilers, and therefore we assessed the efficiency of providing *LP*256 both via silage inoculated with the strain and via direct supplementation in the drinking water. Concurrently, impact of haylage as a forage similar to silage but without any inoculum was tested. The effects of the treatments were evaluated on slow-growing (birds used in organic production) and fast-growing (conventional production) broilers in two separate trials under *C. jejuni* challenge.

## 2. Materials and methods

### 2.1. Experimental design and housing

The experimental setting was previously described in detail by Valečková et al. (2020); a brief description follows. Two experiments were conducted concurrently at the Swedish Livestock Research Centre of the Swedish University of Agricultural Sciences, with approval from the Uppsala region’s animal ethics committee (approval number 5.8.18-16271/2017). The experiments were conducted using fast-growing broilers Ross 308 (R-308), used in conventional production in Sweden, and the Rowan Ranger broilers (RR), with a slower growth preferred in, e.g., organic broiler production. In Experiment 1, a total of 160 unsexed day-old R-308 broiler chickens were used for the 42-day (6-week) experiment, which is considered a normal period of growth for fast-growing strains in the EU. In Experiment 2 a total of 160 unsexed day-old RR broiler chickens (also referred to as “slow-growing broiler”) were used in the 63-d experiment (9 weeks) corresponding to the age at which this broiler type is generally slaughtered in organic production systems in Sweden. In each study, broilers were randomly distributed in groups of eight individuals in 20 raised pens with four dietary treatments and five pen replicates for each treatment, arranged in a randomized block design. In both studies, two random broilers per pen were chosen as focal birds, representatives of the entire pen population. Focals were later on used for the collection of fecal droppings for *C. jejuni* quantification by agar plate culture and at the end of the experiment for cecal content sampling for a quantitative real-time polymerase chain reaction (qPCR) assay to quantify *C. jejuni* loads and for microbiota analysis done by 16S rRNA sequencing.

The experiments were performed in parallel during the winter in an insulated stable equipped with the facilities for automatic control of temperature and light. Each pen had a floor covered by fresh wood shavings and was equipped with a metal feeder and a 3-liter bell drinker.

### 2.2. Experimental diets

Detailed diet specification and forage preservation are stated in Valečková et al. (2020). In brief, fresh feed and water (including treatments) were provided directly after the broilers’ arrival and supplied daily. All experimental diets were based on organic compound feed (13 MJ/kg metabolizable energy and 230 g/kg DM crude protein) and the daily requirement of pellets in all treatment groups was estimated (based on production performance objectives) to ensure *ad libitum* provision. Broilers were assigned to four different treatment groups: silage, haylage, *LP*256, or control. Haylage treatment was included in the study as a forage similar to silage but without any inoculum. Silage and haylage experimental diets were composed as total mixed rations (TMR) containing 85% of pellets and 15% of respective forage (on a DM basis). Additionally, the *LP*256 and the control groups received the organic pelleted compound feed (no forage provided). The *LP*256 group had drinking water inoculated with *L. plantarum* 256 (10^7^ CFU/mL).

Second-cut grass, with a seeding composition of 70% timothy and 30% meadow fescue, was used for the production of forages. Silage was inoculated with *L. plantarum* strain 256 during baling, providing an inoculum concentration of 10^8^ CFU per gram fresh matter. Haylage bales were made without inoculum. After 3 months of storage, bales were separately opened, chopped and thereafter ground to 0.5–1 cm particles. Forage was afterward vacuum-packed (1 kg per bag) and bags were stored at a temperature below 0°C to maintain a similar feed quality throughout the experiments. Enumeration of epiphytic LAB on silage was performed in duplicates monthly (January, February, and March) during the trial period and the pH of silage juice was measured prior to the trials.

### 2.3. Bacterial strains and culturing conditions

Bacterial strains used in this study include *L. plantarum* 256 and *Campylobacter jejuni* #65. The *L. plantarum* strain (also known as *L. plantarum* NC7, Cosby et al., 1989) was previously used in our *in vitro* experiments and proved among other LABs to elicit the best inhibitory effect against *C. jejuni* #65 (unpublished data). The *L. plantarum* strain 256 isolate was stored at – 80°C in Luria Bertani (LB) broth with 20% glycerol. It was propagated in De Man, Rogosa and Sharpe broth for 24 h at 37°C for silage preparation and as a prophylactic probiotic in the study. *C. jejuni* #65 (ST-104, in ST-21 CC; isolated from a broiler chicken in the UK 2006) was cultured in Brucella broth at 42°C under microaerobic (85% N2, 10% CO2, 5% O2) conditions for 24 h. After 24 h incubation, optical density at 405 nm and plate counts were used to determine the infection dose used in the *C. jejuni* challenge.

### 2.4. *Campylobacter jejuni* colonization and quantification

**Figure 1:**
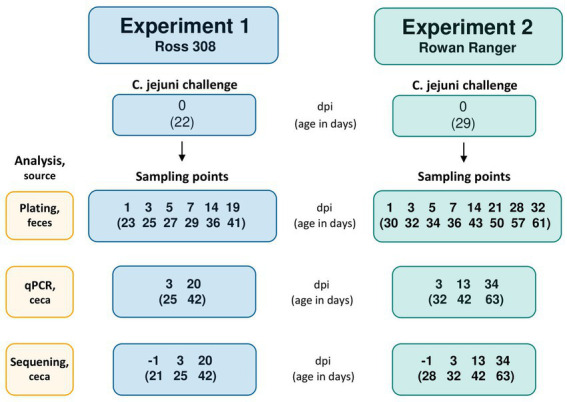
Flowchart with the *Campylobacter jejuni* challenge occasion and following sampling points in Experiment 1 (R-308) and Experiment 2 (RR). The challenge occasion and sampling points are presented as days post-infection (dpi), with the corresponding age of the birds (in days) indicated in brackets.

To investigate the effects of the *L. plantarum* treatments on *C. jejuni* colonization of the broilers’ ceca, all birds were orally challenged (also referred to as “infected”) at 22 d of age in Experiment 1 and 29 d of age (corresponding to the 4 weeks of age at which organic broilers in Sweden must have access to an outdoor environment) in Experiment 2 (Figure 1). On the day of the challenge, 0.5 L of water with 10^6^ CFU/mL of the *C. jejuni* strain #65 was provided in the bell drinker of each pen. The inoculated water was administered for 3 h and *C. jejuni* viability in the water was determined by colony counts on blood agar plates at the start and end of the challenge.

The colonization pattern of *C. jejuni* was monitored during 19 days and 32 days for R-308 and RR, respectively, by fecal culture and colony counts on modified Charcole Cefoperazone Deoxycholate (mCCDA) agar plates. For fresh fecal sampling, two focal birds from each pen were placed individually in plastic boxes. Sterile plastic loops were used for the collection of droppings from the box bottom. Fecal samples were collected from all pens 1 day before the challenge with *C. jejuni*, to verify that the broilers were *Campylobacter* negative before the challenge. One hundred mg of fresh fecal droppings from the focal birds in each pen were collected in 1 mL LB medium supplemented with 20% glycerol on 1, 3, 5, 7, 14, and 19 days post-infection (dpi) in Experiment 1 and 1, 3, 5, 7, 14, 21, 28, and 32 dpi in Experiment 2 (Figure 1). Tubes were directly transported on ice to the laboratory for analysis. Samples were vortexed and centrifuged (100 × *g* for 15 s) to pellet crude fecal matter. Next, 100 μL was withdrawn and serially diluted in 10-fold dilution series. Afterward, 100 μL was plated on mCCDA and incubated for 26 h at 42°C under microaerobic conditions (Campygen, Thermo Fisher). After incubation, colonies were counted on the plate corresponding to the dilution that gave approximately 100 CFU per plate. Raw plate counts data are provided in Supplementary Figure 1 (Experiment 1) and Supplementary Figure 2 (Experiment 2).

### 2.5. Cecal samples collection

In both experiments, one random bird per cage was sacrificed 1 day before infection (−1 dpi) and 3 days after infection (3 dpi) by an intravenous injection of sodium pentobarbital through the wing vein; the birds age corresponding to mentioned dpi is stated in Figure 1. The cecal content was sampled with an aseptic procedure into 2.0 mL screw cap microtubes (Sarstedt AG & Co, Germany) and placed in liquid nitrogen (followed by storing at – 80°C until analyzed). At 42 days of age, all focal birds in Experiment 1 (20 dpi), and one random bird in each replicate in Experiment 2 (13 dpi) were sacrificed and sampled. Experiment 2 focal birds were sacrificed at 63 days of age (34 dpi) followed by cecal sampling as described above. Samples were analyzed by a qPCR assay to assess *C. jejuni* colonization and the microbial composition of cecal content was investigated by 16S rRNA amplicon sequencing.

### 2.6. DNA extraction and qPCR-based *Campylobacter jejuni* quantification

For quantification of *C. jejuni* load in the broiler cecum using qPCR, a standard curve was developed as a reference for the proceeding analysis (Supplementary Table 1). Bacterial colonies of *C. jejuni #65* from a 36-h incubated mCCDA culture were suspended in 300 μL of phosphate-buffered saline (PBS). The suspension was briefly vortexed and divided into 100 μL duplicates, and was diluted in a 10-fold series of PBS for CFU counting on mCCDA plates and incubated under microaerobic conditions for 24 h at 42°C.

Simultaneously, another 100 μL replicate was extracted for DNA and qPCR analysis. The sample was mixed with 200 mg of 0.1 mm zirconia/silica beads (Biospec products, Bartlesville, USA) and 900 μL of ASL lysis buffer (Qiagen, Germany), briefly vortexed, and incubated at 95°C for 5 min to lyse cells, followed by immediate placement on ice for 10 min. The sample was then bead-beaten on Precellys24 sample homogenizer (Bertin Technologies, Montigny-le-Bretonneux, France) at 8000 rpm for 2 × 60 s with 30 s pause to disrupt bacterial cell walls mechanically. Centrifugation of the sample at 2500 x *g* for 1 min followed, and 200 μL of the supernatant was withdrawn into the sample tube together with 20 μL of proteinase K for DNA extraction. Extraction was performed on an EZ1 Advanced XL instrument (Qiagen, Germany) according to the manufacturer’s instructions. The extract was diluted in a 10-fold series of nuclease-free water and used as a template in the real-time PCR for generating a standard curve.

A real-time PCR targeting the d65_1178 gene, specific to *C. jejuni* Strain #65 and its ST type ST-104 (ST-21 CC) was conducted using a primer pair adapted from Atterby et al. (2018). The PCR was performed on a CFX96 Optics Module C1000 Touch Thermal Cycler (Bio-Rad Laboratories, USA). The reaction mixture contained: 1 x SsoAdvanced Universal SYBR Green Supermix, 0.3 μL of forward and reverse primer each, and 1 μL of the template. Reactions were run in triplicates. The amplification parameters were as follows; 98°C for 3 min, 40 cycles of 98°C for 15 s and 63°C for 60 s and followed by a melt curve ranging from 65 to 95°C as a check for assay specificity. Generated qPCR data was analyzed on Bio-Rad CFX Manager 3.1 software (Bio-Rad Laboratories, USA) and Microsoft Excel. Raw qPCR data are provided in Supplementary Figure 3 (Experiment 1) and Supplementary Figure 4 (Experiment 2). The amplification efficiency of the PCR reaction was 76% with R^2^ of 0.9996.

All the cecal samples followed the same pre-treatment, DNA extraction procedure as the *C. jejuni #65* suspension, with minor pre-treatment modifications. Modifications included; 400 μL of ASL lysis buffer, added to the sample and vortexed briefly to homogenize. Then, 120 μL of the sample was used in downstream steps. Sample DNA extracts were analyzed by qPCR and sequencing. For quantification, DNA extracts were run on qPCR along with a standard (*C. jejuni #65*) DNA extract. The CT values obtained from sample runs were compared to that of the standard and transformed into CFU using the generated standard curve equation. The generated CFU was multiplied by five to compensate for a five times dilution of the sample performed during pre-treatment, a dilution not performed on the standard suspension. The ultimate quantification was expressed in CFU/ml by multiplication of 10.

### 2.7. 16S rRNA sequencing

One hundred forty cecal sample DNA extracts were sequenced using the Illumina Miseq PE 250 sequencing platform at Novogene Bioinformatics Technology Co., Ltd. (Beijing, China). The 16S rRNA gene V3–V4 regions were amplified using Illumina primer set 341F (CCTAYGGGRBGCASCAG) and 806R (GGACTACNNGGGTATCTAAT) with a barcode. All template DNAs were normalized to the same concentration. PCR reactions were performed with Phusion® High-Fidelity PCR Master Mix (New England Biolabs, USA). PCR products were separated by electrophoresis on 2% agarose gel, purified with a Qiagen Gel Extraction Kit (Qiagen, Germany) and pooled at equal concentrations. Sequencing libraries were generated using NEBNext Ultra DNA Library Prep Kit (Illumina, USA) following the manufacturer’s recommendations, and index codes were added. Library quality was assessed on the Qubit 2.0 Fluorometer (Thermo Fisher Scientific, USA) and the Bioanalyzer 2100 system (Agilent Technologies, USA).

### 2.8. Sequence analysis

The raw sequencing data were uploaded to the National Center for Biotechnology Information database (NCBI) with accession number PRJNA876811. The bioinformatics data processing was performed by Quantitative Insights into Microbial Ecology 2 – QIIME2 (version 2020.2.0) (Bolyen et al., 2019). The barcode and primer sequence of raw demultiplexed reads were trimmed off and further processed by DADA2 to denoise and dereplicate reads, merge pair-end reads and remove chimeras (Callahan et al., 2016). The truncation length of 221 bp was used for both forward and reverse reads. The phylogenetic tree was built using FastTree and MAFFT alignment (Katoh et al., 2002; Price et al., 2010). The SILVA SSU Ref NR 99132 dataset was first trimmed to the corresponding primer region and trained as a classify-sklearn taxonomy classifier (Pedregosa et al., 2012; Quast et al., 2013; Bokulich et al., 2018). Subsequently, the amplicon sequence variants (ASV) were assigned taxonomy using the resulting classifier. After trimming and quality filtering, the sequencing of 16S rRNA gene yielded a total of 4,539,867 sequences from 140 samples. The ASV table was rarefied according to the minimum reads per sample (i.e., 21,377 reads) (Weiss et al., 2017). The generalized UniFrac distance matrix (alpha = 0.5) and alpha rarefaction was generated using the QIIME2 diversity plugin (Chen et al., 2012; Bolyen et al., 2019).

### 2.9. Statistical analysis

The data generated from plate counts and qPCR from both experiments were organized in Microsoft Corporation (2018) and statistically analyzed in SAS version 9.4 (SAS Institute Inc, 2013). Plating data are graphically represented as scatter panel plots showing bacterial counts as log (CFU/ml) and qPCR data as a box plot; plots were generated with R and the package ggplot2 (Wickham, 2016; R Core Team, 2021). Data from Experiments 1 and 2 were treated by the same pattern.

Statistical analyses of fecal plate count data were performed with a mixed effect linear model (Proc Mixed procedure in SAS) due to the repeated measure structure of the data. The model included treatment, days post-infection (dpi), and their interactions as fixed factors and pen as a random factor. To account for the repeated structure when several observations were made on the same birds (focals) at different dpi we included an error term with an unstructured covariance matrix. Post-hoc tests were conducted to compare *C. jejuni* load (log CFU/ml) at individual dpi among treatment groups as well as to compare all dpi within each treatment. In order to compare *C. jejuni* loads through the whole challenge period between four treatment groups, plate count data were expressed as the mean of all observed dpi samples within one treatment (colonization mean). qPCR data were analyzed with the same mixed-effect linear model and in the same pattern as the plating data. However, the pen as a random factor and repeated structure were removed from the model since only one cecal sample per pen was analyzed. Residual plots were inspected to ensure that residual were approximately normally distributed with equal variances for all models. Results are considered significant if *p* < 0.05.

For cecal microbiota, diversity analyses were performed with the q2-diversity plugin. The rarefied ASV table was used to calculate the number of observed ASV. Kruskal–Wallis rank test with Benjamini & Hochberg (B-H) correction was used to observe statistical differences in a number of observed ASV between groups (i.e., dpi and treatment, Kruskal and Wallis, 1952; Benjamini and Hochberg, 1995). Principal coordinate analysis was used to visualize the difference in the microbial composition based on the generalized UniFrac distances. Permutational multivariate analysis of variance (PERMANOVA) test of generalized UniFrac distance matrix with (B-H) correction was conducted to evaluate the difference among groups (Anderson, 2001). To identify bacterial taxa that differed in abundance between groups, we performed an analysis of composition of microbiomes (ANCOM) (Mandal et al., 2015).

## 3. Results

### 3.1. Higher LAB concentration and lower pH in silage than in haylage

Monthly enumeration of LAB during the experiment period revealed that silage contained 8.0, 7.4, and 7.2 log CFU/g of LAB, respectively, while haylage levels were 5.0, 3.8, and 3.0 log CFU/g. Consequently, silage contained ≥3 × 10-log (CFU/g) higher LAB concentrations than haylage and a gradual decrease in LAB concentrations was observed in both matters. The silage pH measurement just prior to the experiment showed pH 4.4 while haylage displayed pH 6.2.

### 3.2. *Campylobacter jejuni* colonization impacted by silage and haylage treatment in R-308 but not in RR as determined by culture, no significant treatment effects determined by qPCR

Culture results revealed *Campylobacter jejuni* negativity in all birds prior to the infection and successful *C. jejuni* colonization in both broiler types after the challenge. In both experiments, *C. jejuni* loads peaked within 3 days after the challenge and thereafter colonization intensity had decreasing tendency with time.

**Figure 2:**
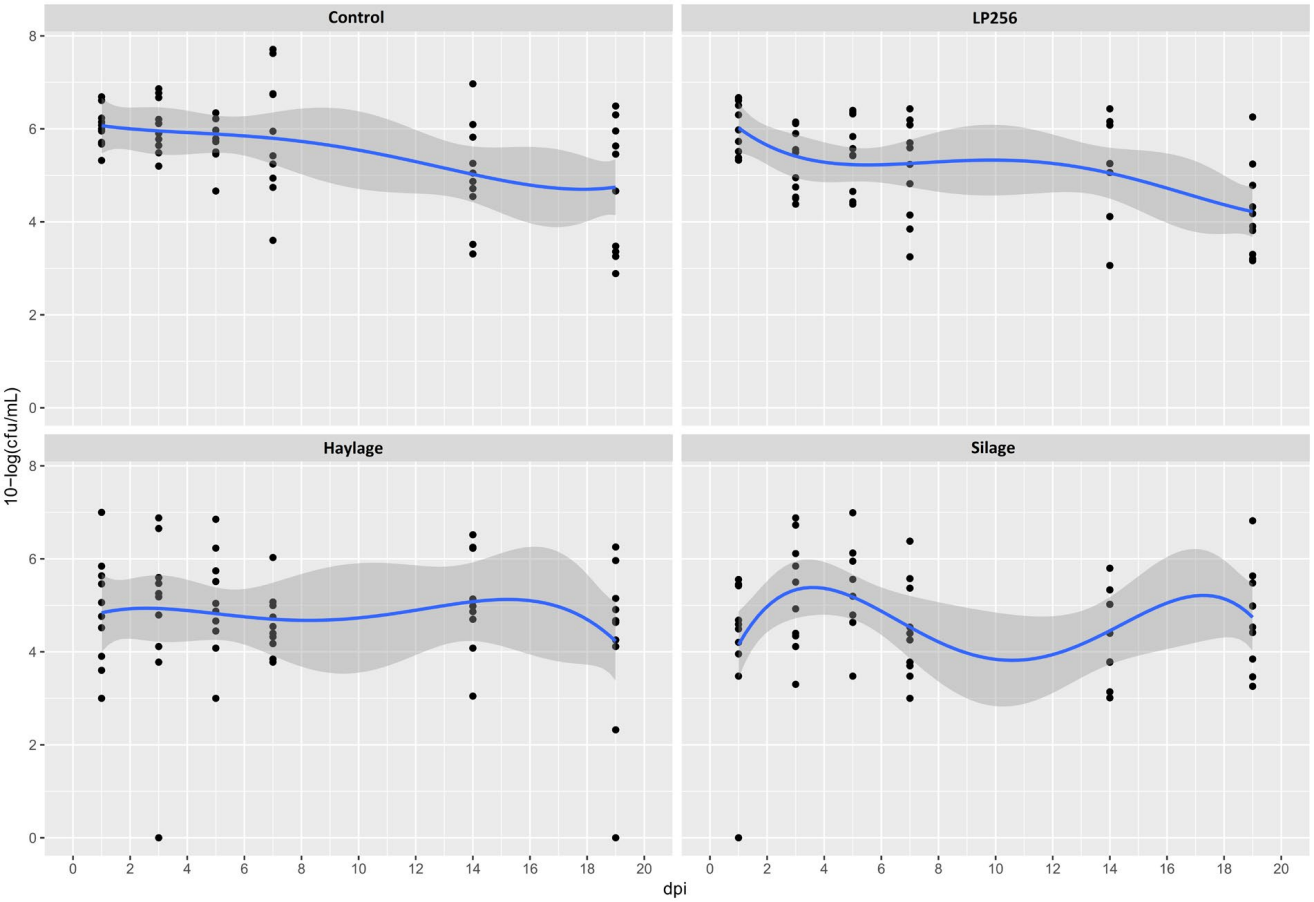
Culture-based colonization patterns of *C. jejuni* in four dietary treatment groups in Experiment 1. Black dots (data points) represent 10-log (CFU/ml) in individual fecal samples at a given day post-infection (dpi). The blue line is a smooth curve representing the trend of colonization based on the mean 10-log (CFU/ml) in each treatment with a 95% confidence band.

In R-308, there was an overall significant treatment effect (*p* = 0.023) observed. Specifically, a significantly lower *C. jejuni* colonization mean was observed in the silage (*p* = 0.010) and haylage (*p* = 0.013) groups in comparison to the control (Figure 2). At 1 dpi, colonization in the silage group was significantly lower (2.01 logs) in comparison to the control group (*p* = 0.039). However, at the end of the experiment (19 dpi), there was no significant difference in the colonization between any of the treatments. No significant effect of *LP*256 (directly provided via the drinking water) treatment on *C. jejuni* loads was observed. A comparison of *C. jejuni* colonization within each treatment at 1 and 19 dpi (start and end of colonization period) revealed no significant difference in bacterial load (CFU/ml). The same was true for colonization comparison between the 3 dpi (supposed peak of *C. jejuni* load) and 19 dpi.

**Figure 3:**
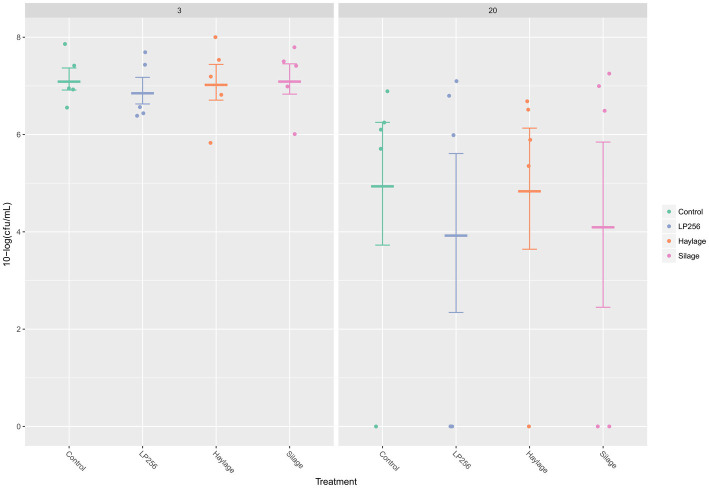
Quantitative PCR-based colonization dynamics of *C. jejuni* in the four dietary treatment groups at 3 and 20 days post-infection (dpi) in Experiment 1. The dots represent *C. jejuni* load (10-log CFU/ml) in individual caecal samples and line bars represent the mean 10-log CFU/ml with standard deviation.

As determined by qPCR (Figure 3), *C. jejuni* loads in ceca at 3 dpi (25 days of age) or 20 dpi (42 days of age) were not significantly affected by dietary treatments in R-308. A comparison of *C. jejuni* colonization within treatment revealed a decreasing pattern between 3 dpi and 20 dpi. However, the differences in *C. jejuni* CFU/ml were not statistically significant.

**Figure 4:**
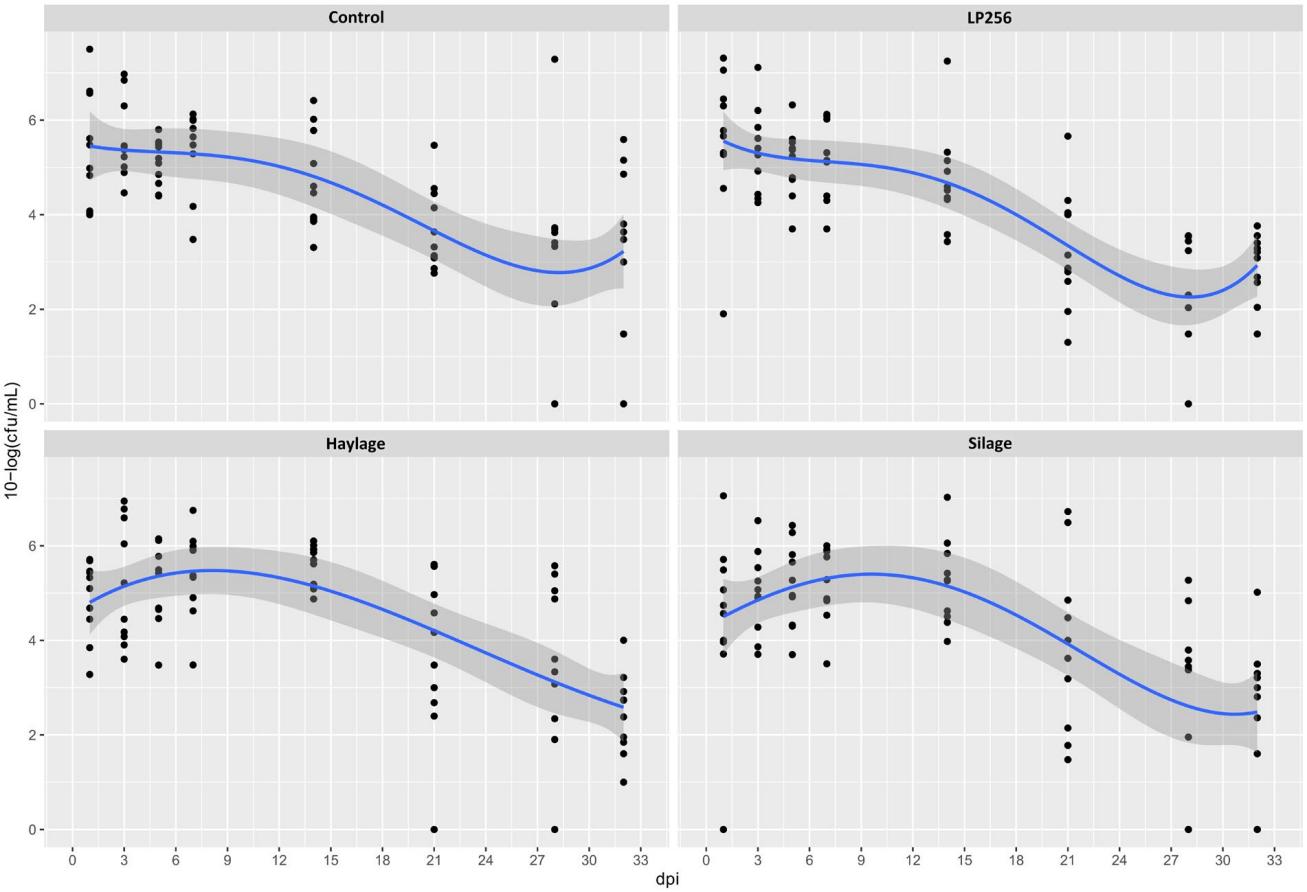
Culture-based colonization patterns of *C. jejuni* in four dietary treatment groups in Experiment 2. Black dots (data points) represent 10-log (CFU/ml) in individual fecal samples at a given day post-infection (dpi). The blue line is a smooth curve representing the trend of colonization based on the mean 10-log (CFU/ml) in each treatment with a 95% confidence band.

No significant effect of treatments on *C. jejuni* loads was observed in RR as determined by culture (Figure 4). However, colonization comparison between the start of the infection period (1 dpi) and end of the trial (32 dpi) within each treatment revealed significant changes with mean reductions in *C. jejuni* of 2.65 and 2.46 10-log (CFU/ml) for *LP*256 and haylage, respectively (*p* = 0.006 and *p* = 0.017, respectively). Colonization comparison between the supposed peak of bacterial load (3 dpi) and end of the trial (32 dpi) displayed significant *C. jejuni* reduction in all treatment groups; *p* = 0.002, *p* = 0.001, *p* = 0.001, and *p* = 0.001 for control, *LP*256, haylage and silage group, respectively.

**Figure 5:**
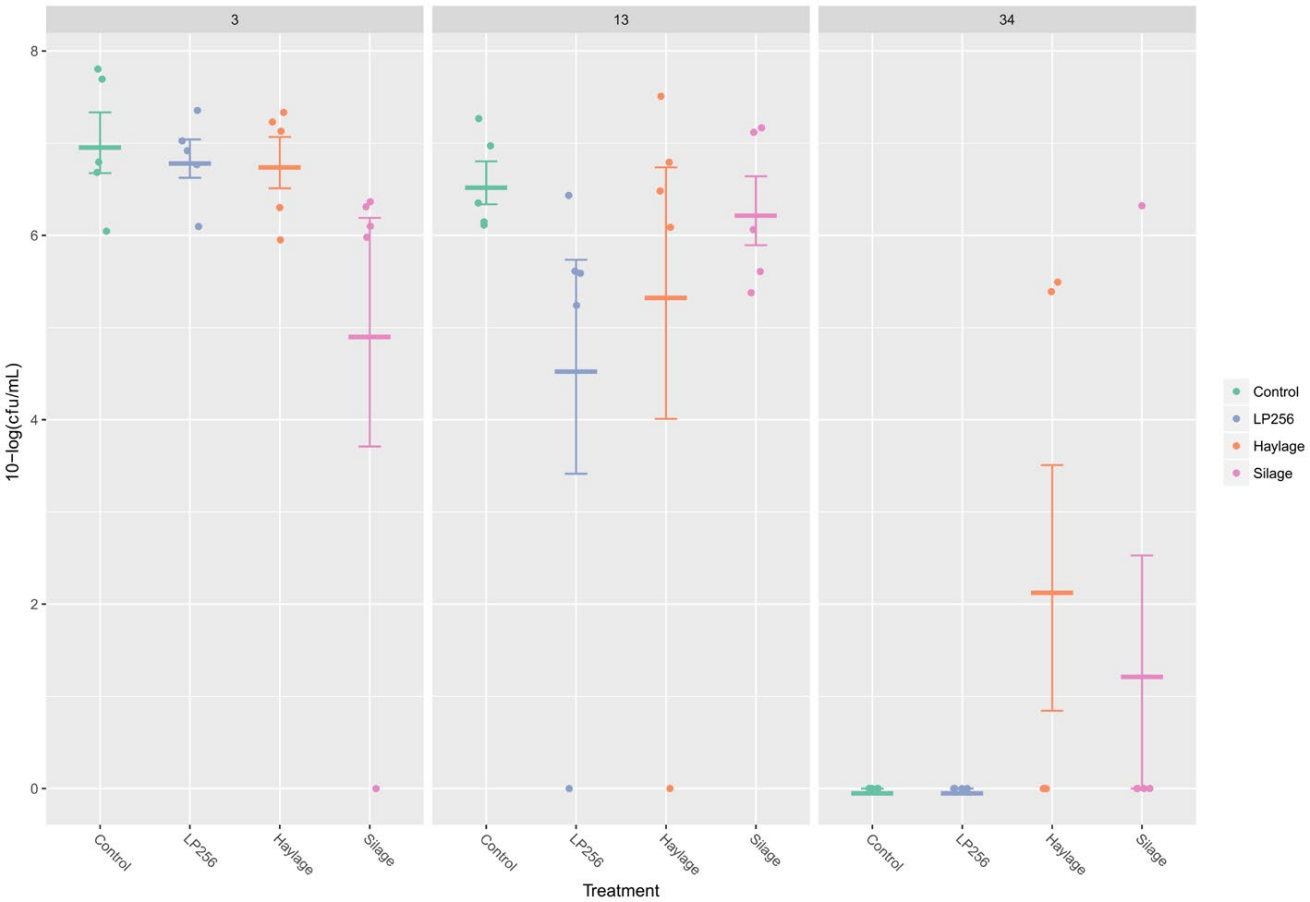
Quantitative PCR- based colonization dynamics of *C. jejuni* in the four dietary treatment groups at 3, 13, and 34 days post-infection (dpi) in Experiment 2. The dots represent *C. jejuni* load (10-log CFU/ml) in individual caecal samples and line bars represent the mean 10-log CFU/ml with standard deviation.

At 3, 13, or 34 dpi, no significant treatment effects on the ceca *C. jejuni* loads were observed in RR as determined by qPRC (Figure 5). Comparing *C. jejuni* loads between the supposed peak of bacterial colonization (3 dpi; 34 days of age) and end of the trial (34 dpi; 63 days of age) revealed significant reductions in control, *LP*256, and haylage group (*p* = 0.001, *p* = 0.001, and *p* = 0.024, respectively). A comparison between cecal *C. jejuni* colonization at 13 dpi (42 days of age) and 34 dpi for each treatment displayed significant *C. jejuni* reductions in the control, *LP*256, and silage groups (*p* = 0.001, *p* = 0.032, and *p* = 0.014, respectively).

### 3.3. *Firmicutes* and *Bacteroidota* dominated both R-308 and RR cecal microbiota, with significant changes observed in their relative abundances after the *Campylobacter* challenge

Characterization of the cecal microbiota composition before and after the *C. jejuni* challenge in Experiments 1 and 2 was performed by 16S rRNA amplicon sequencing. A total of 140 samples were analyzed and altogether 675 amplicon sequence variants (ASVs) were identified, representing 122 taxonomic genera, 52 families, 33 orders, 13 classes, and 5 phyla. The rarefaction curves of observed ASVs revealed sufficient sequencing depth to capture species richness at all time points tested in Experiment 1 (Supplementary Figure 5) and Experiment 2 (Supplementary Figure 6).

**Figure 6:**
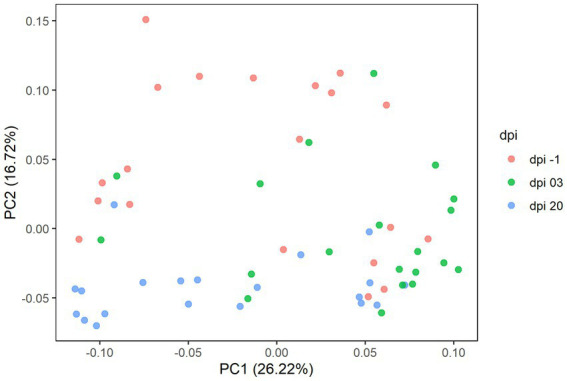
Principal coordinate analysis (PCoA) plot showing the differences in beta diversity based on generalized UniFrac distances between samples at −1, 3, and 20 days post-infection (dpi); represented by 20 caecal samples, respectively, for R-308. The dpi −1 is represented by samples before infection.

In detail, 619 ASVs, representing 109 taxonomic genera and 5 phyla were observed in Experiment 1. Results of the principal coordinate analysis (PCoA) are shown in Figure 6 to visualize the variation of cecal microbiota between different days post-infection. Significant differences in the gut microbiota composition between different dpi were observed (*p* = 0.001), while no clear effect of the feed treatments on cecal microbiota composition was found; therefore, the treatments were pooled for further analysis.

**Figure 7:**
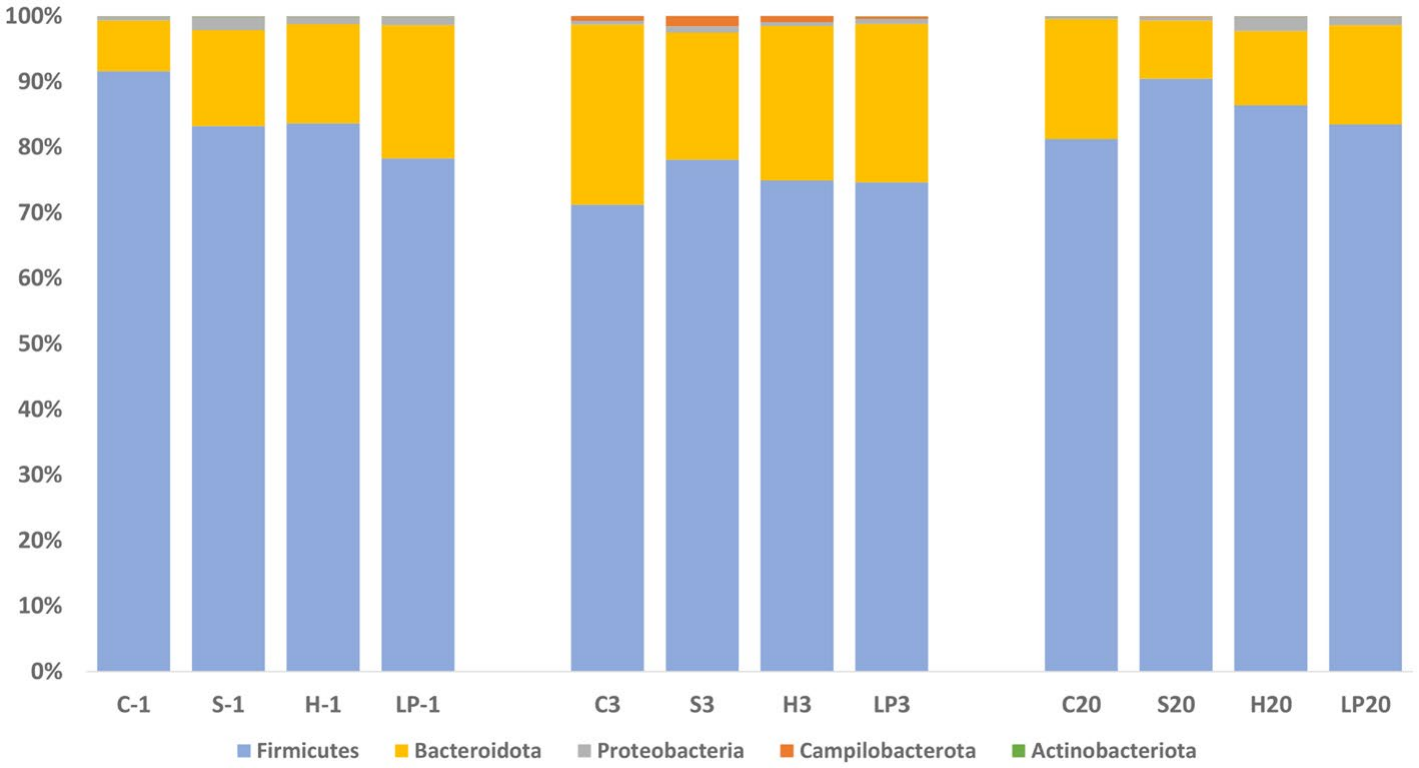
Phylum-level relative abundance (%) of broiler caecal microbiota in Experiment 1 (R-308) at each treatment and days post-infection (dpi): −1, 3, and 20. The treatment groups shown are the following: dietary treatment and dpi. C, control feed; S, diet based on 85% of pellets and 15% of silage; H, diet based on 85% of pellets and 15% of haylage; LP, control feed and water inoculated with 10^7^ CFU/mL of viable *L. plantarum* 256.

At the phylum level, *Firmicutes* and *Bacteroidota* comprised more than 97.5% of the bacteria’s relative abundance (RA) in all treatment groups at −1, 3, and 20 dpi, suggesting that they were the major components of the cecal microbiota (Figure 7). Since the different dietary treatments had no influence on the gut microbiota composition, treatments were pooled together and changes between different days post-infection were investigated. Changes in the RA at phylum level was observed after the *Campylobacter* challenge; a temporary decrease in the RA of phylum *Firmicutes* (mean RA at −1, 3, and 20 dpi was 84.2, 74.7, and 85.4% respectively) was substituted by a corresponding significant increase in *Bacteroidota* (mean RA at −1, 3, and 20 dpi was 14.5, 23.7, and 13.4%, respectively). The RA of phylum *Proteobacteria* decreased at 3 dpi and returned to a similar level as before the *C. jejuni* challenge at 20 dpi (mean RA at −1, 3, and 20 dpi was 1.3, 0.7, and 1.1% respectively). A significant increase in the RA of phyla *Campilobacterota* was observed at 3 dpi (mean RA of *Campilobacterota* at −1, 3, and 20 dpi was 0.01, 0.9, and 0.1% respectively). The RA of *Actinobacteriota* ranged from 0.02 to 0.04%.

At the genus level, the top 25 genera (Table 1) constituted 89% of the total sequencing read pool. Genus *Campylobacter* was added as the 26th genus due to the interest of this study. A description of major changes in the RA at the genus level between different dpi follows: genus *Bacteroides* was the most dominant in the cecal microbiota at −1 dpi and clearly most dominant after the *C. jejuni* challenge (3 dpi). Thereafter, a considerable decrease was observed to the advantage of other genera at 20 dpi (Table 1). *Clostridia* UCG-014 and uncultured *Ruminococcaceae* continuously increased in RA throughout the sampling points; where *Clostridia* UCG-014 became the most abundant genus at 20 dpi. A significant decrease in the RA of the twelfth most abundant genus *Lactobacillus* was observed after the *C. jejuni* challenge at 3 dpi. However, at 20 dpi the RA of this genus reached higher levels than before *C. jejuni* challenge. In the genera *Clostridia* vadinBB60 group and unclassified *Lachnospiraceae*, a decrease in RA appeared at 3 dpi and remained at a similar level at 20 dpi. The RA of the genera *Faecalibacterium* (the second most dominant bacterial genus in broilers’ ceca), *Eisenbergiella*, *Subdoligranulum*, and *Escherichia-Shigella* decreased after *C. jejuni* challenge (3 dpi) but eventually, an increase was observed in all four genera at 20 dpi. As expected, the RA of genus *Campylobacter* significantly increased after the challenge (3 dpi) but had diminished at 20 dpi. Although clear general trends in the mean RA of different genera could be observed over the experiment as described above, there were high individual variations in the birds’ cecal microbiota composition within the same treatment group at each infection time point (dpi).

**Table 1:** The mean and SD of relative abundance (%) of the top 25 genera of broiler cecal microbiota at each day post-infection (dpi) in Experiment 1.

Family	Genus	−1 dpi (%)	3 dpi (%)	20 dpi (%)
Bacteroidaceae	*Bacteroides*	14.5 ± 5.2	23.7 ± 3.3	13.4 ± 4.2
Ruminococcaceae	*Faecalibacterium*	9.4 ± 4.0	8.2 ± 3.9	13.7 ± 1.5
Same as genus	*Clostridia* UCG-014	4.1 ± 0.7	9.9 ± 1.9	16.1 ± 3.4
Same as genus	*Clostridia* vadinBB60 group	12.7 ± 5.3	6.8 ± 2.7	6.4 ± 0.6
Lachnospiraceae	unclassified *Lachnospiraceae*	8.2 ± 1.6	6.9 ± 0.5	6.3 ± 0.9
Lachnospiraceae	*Ruminococcus torques* group	6.2 ± 0.9	6.7 ± 1.4	5.4 ± 0.9
Lachnospiraceae	*Eisenbergiella*	3.7 ± 0.9	2.6 ± 0.3	2.7 ± 0.6
Ruminococcaceae	*Subdoligranulum*	2.0 ± 1.3	1.8 ± 0.7	3.5 ± 1.1
Ruminococcaceae	*Negativibacillus*	2.4 ± 0.2	2.0 ± 1.2	2.1 ± 0.2
Oscillospiraceae	*Colidextribacter*	2.2 ± 0.3	2.5 ± 0.5	1.6 ± 0.2
Ruminococcaceae	uncultured *Ruminococcaceae*	1.6 ± 0.1	1.8 ± 0.2	2.2 ± 0.4
Lactobacillaceae	*Lactobacillus*	1.7 ± 0.7	0.9 ± 0.9	2.9 ± 1.2
Oscillospiraceae	uncultured *Oscillospiraceae*	1.3 ± 0.3	2.1 ± 0.4	1.8 ± 0.5
Lachnospiraceae	*Lachnospiraceae* NK4A136 group	2.6 ± 0.7	1.2 ± 0.2	1.4 ± 0.5
Peptostreptococcaceae	unclassified *Peptostreptococcaceae*	4.3 ± 7.6	0.1 ± 0.2	0.0 ± 0.0
Same as genus	[*Eubacterium*]_coprostanoligenes_group	1.4 ± 0.2	1.2 ± 0.1	1.6 ± 0.1
Lachnospiraceae	*Lachnospiraceae* GCA-900066575	1.4 ± 0.3	1.2 ± 0.4	1.1 ± 0.4
Oscillospiraceae	unclassified *Oscillospiraceae*	1.1 ± 0.3	1.9 ± 0.7	0.5 ± 0.3
Oscillospiraceae	*Oscillibacter*	1.2 ± 0.3	1.2 ± 0.1	1.0 ± 0.2
Lachnospiraceae	*Blautia*	1.6 ± 0.5	1.0 ± 0.1	0.8 ± 0.2
Lachnospiraceae	*Lachnoclostridium*	1.5 ± 0.5	1.1 ± 0.2	0.8 ± 0.2
Ruminococcaceae	*Ruminococcaceae* Incertae Sedis	1.0 ± 0.3	1.1 ± 0.3	0.9 ± 0.1
Enterobacteriaceae	*Escherichia-Shigella*	1.2 ± 0.6	0.7 ± 0.2	1.1 ± 0.9
Same as genus	*Bacilli*_RF39	0.6 ± 0.3	1.0 ± 0.2	1.4 ± 0.4
Butyricicoccaceae	*Butyricicoccus*	0.8 ± 0.1	1.0 ± 0.3	0.5 ± 0.2
*Campylobacteraceae	*Campylobacter	0.01 ± 0.01	0.9 ± 0.5	0.1 ± 0.1

Genus *Campylobacter* (*) was added as the 26th genus due to the interest of the study.

**Figure 8:**
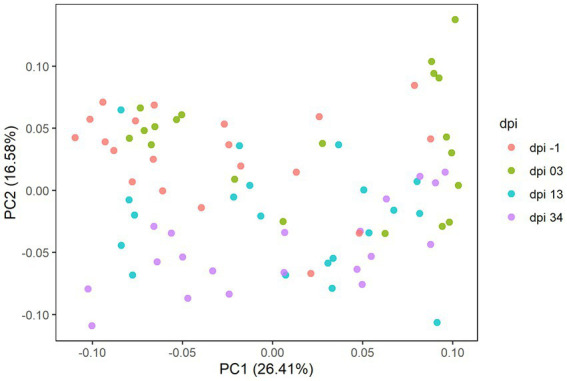
Principal coordinate analysis (PCoA) plot showing differences in beta diversity based on generalized UniFrac distances between samples at −1, 3, 13, and 34 days post-infection (dpi); represented by 20 samples, respectively, for RR. The dpi −1 is represented by samples before infection.

In Experiment 2, sequencing results comprised 671 ASVs, representing 121 taxonomic genera and 5 phyla. The results of the principal coordinate analysis are presented in the PCoA plot (Figure 8), where significant differences in the microbiota composition were observed (*p* = 0.001), while no clear effect of the feed treatments was found.

**Figure 9:**
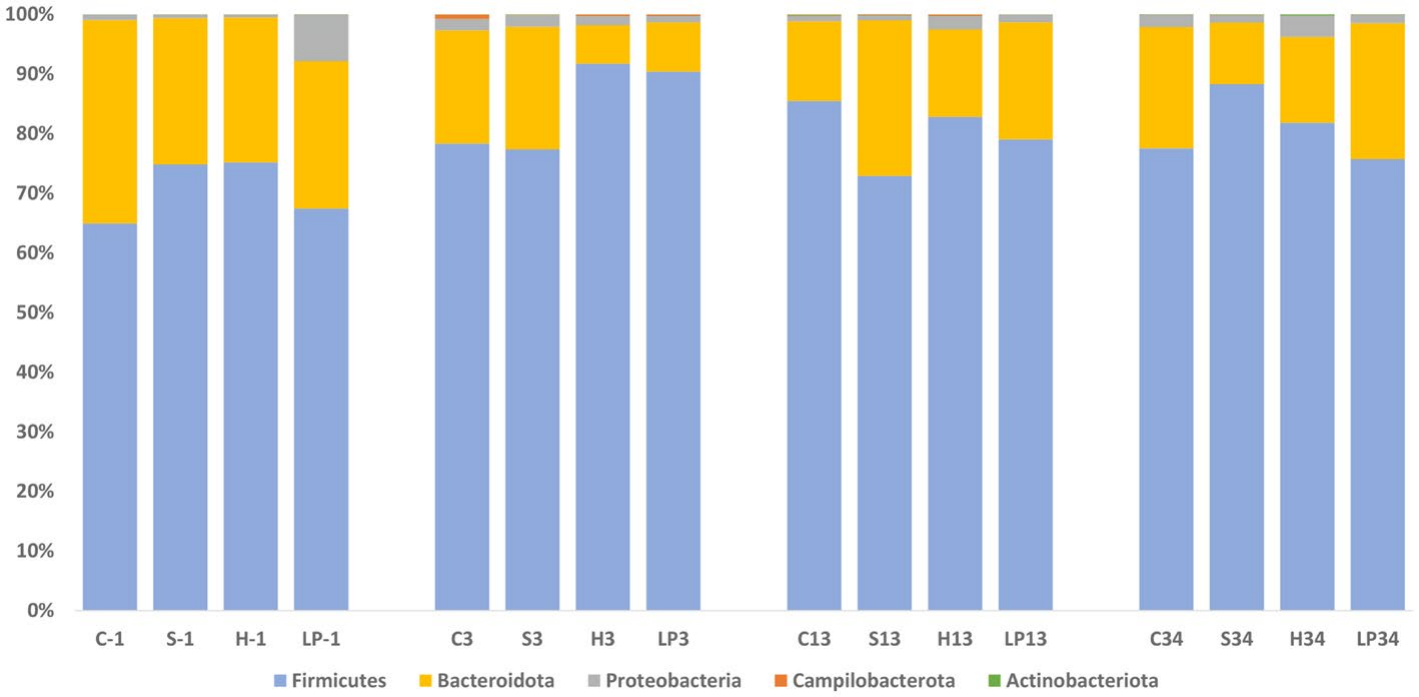
Phylum relative abundance (%) of broiler caecal microbiota in Experiment 2 (RR) at each treatment and days post-infection (dpi): −1, 3, 13, and 34. The treatment groups shown are the following: dietary treatment and dpi. C, control feed; S, diet based on 85% of pellets and 15% of silage; H, diet based on 85% of pellets and 15% of haylage; LP, control feed and water inoculated with 10^7^ CFU/mL of viable *L. plantarum* 256.

As seen in Experiment 1, cecal microbiota composition at the phylum level was dominated by *Firmicutes* and *Bacteroidota* also in Experiment 2; representing together at least 92.2% of the bacteria in all treatment groups (Figure 9) at −1, 3, 13, and 34 dpi. Proportional changes in RA were observed after the *C. jejuni* challenge (dpi 3) by an increase of phylum *Firmicutes* (mean RA at −1, 3, 13, and 34 dpi was 70.6, 84.5, 80.1, and 81.8% respectively) with a concomitant decrease of the taxonomic group *Bacteroidota* (mean RA at −1, 3, 13, and 34 dpi was 26.9, 13.6, 18.5, and 17.0% respectively). This was in contrast to the reverse pattern observed in Experiment 1. The RA of phylum *Proteobacteria* decreased after the infection (3 dpi), remained on a similar level at 13 dpi, and eventually increased again at 34 dpi; mean RA at −1, 3, 13, and 34 dpi was 2.4, 1.6, 1.3, and 2.1%, respectively. A significant increase in RA of the phylum *Campilobacterota* was observed at 3 dpi; mean RA at −1, 3, 13, and 34 dpi was 0.01, 0.3, 0.1, and 0.01%, respectively. The RA of phylum *Actinobacteriota* increased continuously throughout the experiment, ranging from 0.03–0.09%.

The top 25 genera (Table 2) constituted 91% of the total sequencing read pool. Genus *Campylobacter* was added as the 26th genus due to the interest of the study. A description of the trends in relative abundance at genus level during the experiment period follows: *Faecalibacterium* was the second most abundant genus at −1 dpi, the most abundant after the *C. jejuni* challenge (3 dpi), and clearly the most abundant at 13 and 34 dpi. *Bacteroides* dominated the cecal microbiota at −1 dpi. Despite a considerable decrease after *C. jejuni* challenge, the genus was the second most dominant at 3, 13, and 34 dpi. The RA of the genera *Clostridia* UCG-014, *Ruminococcus torques* group, and uncultured *Ruminococcaceae* peaked at 3 dpi, and thereafter gradually decreased at 13 and 34 dpi. Genus *Lactobacillus* was the ninth most abundant bacteria present in the ceca and its RA increased throughout all sampling points. Relative abundance of genera unclassified *Lachnospiraceae* and *Subdoligranulum* increased at 3 dpi, decreased at 13 dpi, and was maintained at a similar level at 34 dpi. In the genus *Clostridia* vadinBB60 group, a continuous decrease in RA was seen throughout the sampling points. *Escherichia-Shigella* decreased after the challenge and remained on the same level at 13 dpi; thereafter an increase was observed at the end of the trial. The RA of *Campylobacter* significantly increased after the infection (3 dpi) and declined at 13 and 34 dpi. As observed in Experiment 1, a high individual variation in birds’ cecal microbiota composition was observed also in Experiment 2.

**Table 2:** The mean and SD of relative abundance (%) of the top 25 genera of broiler cecal microbiota at each day post-infection (dpi) in Experiment 2.

Family	Genus	−1 dpi (%)	3 dpi (%)	13 dpi (%)	34 dpi (%)
Ruminococcaceae	*Faecalibacterium*	14.1 ± 4.8	14.7 ± 2.4	25.9 ± 4.2	27.9 ± 4.6
Bacteroidaceae	*Bacteroides*	25.9 ± 5.8	14.6 ± 8.7	18.5 ± 5.8	17.0 ± 5.7
Same as genus	*Clostridia* UCG-014	6.9 ± 0.9	13.9 ± 3.2	9.5 ± 1.7	7.2 ± 2.6
Lachnospiraceae	unclassified *Lachnospiraceae*	5.9 ± 1.8	6.3 ± 0.4	5.0 ± 0.8	5.6 ± 0.7
Ruminococcaceae	*Subdoligranulum*	4.2 ± 1.4	6.1 ± 3.7	2.7 ± 0.7	3.0 ± 1.6
Same as genus	*Clostridia* vadinBB60 group	5.3 ± 1.8	5.0 ± 2.1	3.3 ± 0.6	2.1 ± 1.1
Lachnospiraceae	*Ruminococcus torques* group	4.1 ± 0.2	4.4 ± 0.6	3.6 ± 0.4	3.2 ± 0.7
Lachnospiraceae	*Eisenbergiella*	2.5 ± 0.6	2.1 ± 0.2	2.9 ± 0.9	2.8 ± 0.8
Lactobacillaceae	*Lactobacillus*	1.1 ± 0.5	2.0 ± 0.6	2.6 ± 0.4	3.1 ± 1.1
Ruminococcaceae	*Negativibacillus*	2.3 ± 0.1	1.8 ± 0.2	2.1 ± 0.4	2.4 ± 0.6
Ruminococcaceae	uncultured *Ruminococcaceae*	2.0 ± 0.3	2.2 ± 0.5	2.0 ± 0.3	1.9 ± 0.5
Enterobacteriaceae	*Escherichia-Shigella*	2.8 ± 3.4	1.3 ± 0.6	1.3 ± 0.7	1.7 ± 1.0
Oscillospiraceae	*Colidextribacter*	2.0 ± 0.3	1.8 ± 0.3	1.6 ± 0.4	1.3 ± 0.2
Lachnospiraceae	*Lachnospiraceae* NK4A136 group	1.1 ± 0.9	0.9 ± 0.1	1.7 ± 0.7	2.6 ± 3.3
Same as genus	[*Eubacterium*] coprostanoligenes gr.	1.6 ± 0.2	2.3 ± 0.6	1.5 ± 0.3	0.9 ± 0.3
Oscillospiraceae	uncultured *Oscillospiraceae*	1.6 ± 0.3	1.5 ± 0.2	1.2 ± 0.3	1.3 ± 0.3
Oscillospiraceae	*Oscillibacter*	1.3 ± 0.3	1.2 ± 0.1	1.1 ± 0.3	1.4 ± 0.3
Same as genus	*Bacilli* RF39	1.0 ± 0.4	1.1 ± 0.5	1.2 ± 0.7	1.4 ± 0.6
Lachnospiraceae	*Lachnoclostridium*	1.1 ± 0.3	1.1 ± 0.2	0.8 ± 0.1	1.2 ± 0.4
Lachnospiraceae	*Lachnospiraceae* GCA-900066575	0.9 ± 0.3	0.8 ± 0.2	0.8 ± 0.2	1.1 ± 1.1
Oscillospiraceae	*Flavonifractor*	0.7 ± 0.1	0.9 ± 0.2	0.7 ± 0.2	1.1 ± 0.3
Erysipelatoclostridiaceae	*Erysipelatoclostridium*	0.6 ± 0.3	1.2 ± 1.0	0.7 ± 0.5	0.4 ± 0.3
Lachnospiraceae	*Blautia*	0.7 ± 0.2	0.8 ± 0.2	0.6 ± 0.1	0.8 ± 0.1
Ruminococcaceae	*Ruminococcus*	0.4 ± 0.1	0.8 ± 0.4	1.2 ± 0.4	0.4 ± 0.2
Ruminococcaceae	*Ruminococcaceae* Incertae Sedis	0.8 ± 0.4	0.8 ± 0.2	0.6 ± 0.2	0.5 ± 0.2
*Campylobacteraceae	*Campylobacter	0.01 ± 0.01	0.31 ± 0.29	0.13 ± 0.09	0.01 ± 0.01

Genus *Campylobacter* (*) was added as the 26th genus due to the interest of the study.

## 4. Discussion

Previous studies have shown that probiotics can modulate broilers’ gastrointestinal microbiota, provide beneficial health effects, and increase birds’ resistance to pathogens (Pourabedin and Zhao, 2015). In particular, LAB have been shown to provide inhibitory effects on *C. jejuni* colonization, and feed additives containing such bacteria could therefore be a promising approach to reduce the occurrence of *Campylobacter* spp. in primary production (Guyard-Nicodème et al., 2016). Although some studies have reported promising effects, there are challenges related to the storage, distribution, and rationally feasible means of administration of such probiotics to the broilers (Krysiak et al., 2021). In this study, we assessed the possibility of providing LAB by the inclusion of grass silage inoculated with the strain *Lactiplantibacillus plantarum* 256 in the broiler’s daily feed. According to Commission Regulation (EC) 889/2008 (2008), all birds kept in organic settings in the EU must have daily access to forage, with grass silage being one of the allowable options. With this requirement in mind, we sought to investigate whether the silage may serve as a diet component with the potential to reduce *C. jejuni* in broilers’ guts. In addition, to evaluate whether the potential effect was caused by *L. plantarum 256* itself, we also tested the direct provision of this strain via drinking water.

Our results from colony counts after agar plate culturing for R-308 (Experiment 1) displayed lower *C. jejuni* mean colonization in the silage and haylage treatment groups and significantly lower *C. jejuni* colonization (2 logs) in the silage group compared to the control 1 day after the challenge. However, this effect did not last and no differences in colonization were observed between treatment groups at the end of the experiment period. This result suggests that silage and to some extent haylage could have an inhibitory effect against low loads of ingested *C. jejuni* in the R-308, but clearly could not protect the broilers from *C. jejuni* colonization. A similar initial inhibitory effect could not be observed in the RR where no differences in *C. jejuni* loads between the treatment groups were observed in Experiment 2. High LAB content and low pH in fermented feeds have been previously reported as promising feed attributes in order to reduce the Ross 308 broiler chickens’ susceptibility to *Campylobacter* spp. colonization (Heres et al., 2004). In the current study, lactic acid bacteria counts in ensiled forage and pH evaluation revealed that silage contained notably higher LAB concentrations and lower pH in comparison to the haylage. This may partly explain why *C. jejuni* loads at the beginning of the challenge in Experiment 1 were significantly lower in the silage group compared to the control, while only a moderate (non-significant) reduction was observed in the haylage treatment.

*In vivo* trials investigating the silage effect on *C. jejuni* colonization are currently rare. A study published by Ranjitkar et al. (2016) showed no significant differences in the load of *C. jejuni* between the treatment groups provided with different levels of crimped kernel maize silage in diets (replacement of 15 and 30% of pelleted feed with maize-silage) in comparison to the control. This discrepancy in results may be explained by differences in the type of silage, consumption levels, or by effects of broiler type and and age. The broiler type and age are also likely explanations as to why the reduction of *C. jejuni* loads were observed at the beginning of Experiment 1 in the R-308, while no effect was observed in the RR in Experiment 2. It is well known that fast-growing birds have a higher feed intake than slow-growing ones (Quentin et al., 2004; Sarica et al., 2020; Jong et al., 2021). For that reason, it was of interest to test the effects of silage in both fast- and slow-growing broilers. Hence, since a numerically higher intake of total mixed ratios, containing forage as silage and haylage, was observed in Ross 308 compared to Rowan Ranger birds (Valečková et al., 2020), it is possible that the inhibitory effect of silage on *C. jejuni* colonization seen at the beginning of Experiment 1 was related to the level of daily consumption of forage.

No significant treatment effect was seen on the reduction of *C. jejuni* in feces or ceca when *Lactiplantibacillus plantarum* 256 was directly provided in the drinking water to the R-308 or RR, neither by plate counts nor by qPCR. This suggests that although extracts from silage inoculated with this strain could inhibit *C. jejuni* growth *in vitro* (unpublished data), the presence of this strain in the broiler’s intestinal tract at the level of administration (10^7^ CFU/mL) used in this study could not effectively inhibit *C. jejuni* colonization. This could be related both to dosage and to the strain used. In a study by Arsi et al. (2015), 26 LAB isolates with the greatest inhibitory activity *in vitro* were further tested in a broiler trial where birds were challenged with 10^4^ CFU *C. jejuni* in a 100 μL suspension of tryptone salt broth. Only 3 out of these 26 isolates demonstrated a reduction in *Campylobacter* counts (approximate 1–2 log) in comparison to the control. *In vitro* assay results are known to not always translate into comparable results under *in vivo* settings, due to differences in the final probiotic supplement composition, its dose and application pattern, trial conditions, and thus different outcomes of the probiotic activity. In addition, *in vitro* studies do not take into account the variability and complexity of the birds’ gastrointestinal environment and their interaction with probiotics and *Campylobacter* strains (Smialek et al., 2021). Nevertheless, inhibitory effects on *C. jejuni in vivo* after direct administration of *Lactiplantibacillus* spp. have been demonstrated in several studies. For example, Saint-Cyr et al. (2017) showed that Ross broilers treated with oral gavage of *Lactobacillus salivarius* SMXD51 (10^7^ CFU) at 24 h after hatch displayed a significant reduction in *C. jejuni* loads present in the gut at 14 days of age (0.82 logs) and 35 days of age (2.81 logs) in comparison to the control. Additionally, Neal-McKinney et al. (2012) reported that *Lactobacillus crispatus* JCM 5810 administered to broiler chickens by oral gavage (10^8^ CFU) at the day of hatch and 4 days post-hatch was an effective competitive exclusion organism for *C. jejuni* resulting in a reduction in the total number of *C. jejuni* colonized broilers and lower microbial load at 21 days post-hatch.

In both experiments in this study, *C. jejuni* loads in feces peaked at the beginning of the challenge, after which a decrease over time was observed. This is in agreement with previous findings, e.g., Achen et al. (1998) found that 70% of the broilers were shedding *C. jejuni* within 48 h after artificial infection, and a steady decline in fecal shedding was observed after the third-week post-infection; at 6 weeks after infection, only 38% of the birds were shedding *C. jejuni* in their feces. In the current study, the decline of *C. jejuni* loads with time was more prominent in Experiment 2 (in the RR), where the comparison between the peak of *C. jejuni* bacterial load at 3 dpi to that at the end of the challenge at 34 dpi, displayed significant *C. jejuni* reduction in all treatment groups. However, this was likely due to the fact that in Experiment 2, *C. jejuni* colonization after the challenge was monitored for 2 weeks longer than in Experiment 1.

*Campylobacter jejuni* loads in cecal samples from both experiments were quantified using a qPCR assay. Comparison between cecal samples analyzed by qPCR and fecal samples analyzed by plate counting revealed higher numbers of *C. jejuni* in cecal samples in both experiments, consistent with previous studies (Berrang et al., 2000; Rudi et al., 2004). However, in some cases, *C. jejuni* loads in cecal samples were below the detection limit of the qPCR, despite fecal cultures being *C. jejuni* positive. This inconsistency can be attributed to the lower sensitivity of our qPCR, whose limit of detection was 3.3 × 10^5^ CFU/g (Appendix 3) and is likely the reason for the significant *C. jejuni* reduction observed in Experiment 2 when different infection time points (3 dpi to 34 dpi and 13 dpi to 34 dpi) were compared. Consistent with the findings from the *C. jejuni* colony counts from fecal samples (except observation in the forage treatments in Experiment 1), there were no significant effects of the dietary treatments on the cecal *C. jejuni* loads in either of the two experiments. Similarly, there was no significant reduction in cecal *C. jejuni* loads between 3 dpi and the end of Experiment 1, but a significant reduction was seen at the end of Experiment 2 in *LP*256, haylage, and silage groups. This reduction seemed to be independent of the treatment, consistent with our culture-based results from fecal samples.

The broiler digestive tract is colonized by a wide variety of bacterial species, with the caecum being by far the most densely colonized and studied microbial site of the gut (Pourabedin and Zhao, 2015). Generally, the major phylum in the broiler cecal microbiota is *Firmicutes*, followed by two less abundant phyla, *Bacteroidota*, and *Proteobacteria* (Oakley et al., 2014; Kers et al., 2018). This is reflected in our current results from Illumina 16S amplicon sequencing of cecal samples at different infection time points throughout the two experiments, where *Firmicutes* was the most abundant phylum, followed by the phylum *Bacteroidota*. Lower relative abundances of *Proteobacteria*, *Campilobacterota* (as a result of the *C. jejuni* challenge), and *Actinobacteriota* were observed. Before the *C. jejuni* challenge (−1 dpi), a lower relative abundance of *Bacteroidota* was observed in all treatment groups in R-308, than its RA in RR. Based on results from previous studies by Connerton et al. (2018) and Richards et al. (2019), this difference could be due to the type of broiler used, but also due to the different ages of the birds at the *C. jejuni* exposure since the RR birds in Experiment 2 were challenged 7 days later that the R-308 in Experiment 1. *Actinobacteria* and *Proteobacteria* together represented less than 2 and 2.5% in R-308 and RR cecal samples, respectively, which aligns with their usual representation of around 2 to 3% in the total broiler cecal microbiota (Rychlik, 2020).

There were no significant effects of the different feed treatments on the broiler’s cecal microbiota composition in either of the two different broiler types. However, several distinct changes in relative abundance related to the *C. jejuni* challenge were noted both on phylum and genus levels. In Experiment 1, a decrease in the RA of *Firmicutes* appeared in the R-308 after the *C. jejuni* challenge, which was accompanied by a parallel increase in the RA of *Bacteroidota*. In Experiment 2, the opposite was observed. Previous studies have reported changes in *Bacteroidota* abundance linked to interactions with *Campylobacter*, where, e.g., elevated RA of *Bacteroidetes* was found in *Campylobacter-positive* broilers (Sofka et al., 2015), in agreement with our observations at 3 dpi in R-308. Interestingly, the opposite correlation was observed in a study by Sakaridis et al. (2018) where the elevated abundance of *Firmicutes* and decreased *Bacteroidetes* levels were found in broilers with high *Campylobacter* counts, in line with our observations in RR after the *C. jejuni* challenge. This shows the complexity of gut microbiota interactions, where one stimulus (*C. jejuni* challenge in this case) may have opposing effects on the microbiota composition in two different broiler types.

In both experiments, the predominant phylum *Firmicutes* largely consisted of class Clostridia represented by the genera *Faecalibacterium*, *Clostridia* UCG-014, *Clostridia* vadinBB60 group, and unclassified *Lachnospiraceae*, with other bacteria belonging to families *Lachnosporaceae* and *Ruminococcaceae* at lower percentages. Phylum *Bacteroidota* on the other hand consisted of the sole genus *Bacteroides*. This genus is able to produce short-chain fatty acids, compounds contributing to maintaining mucosal integrity, immunity, and health of broilers (Ali et al., 2022). The most dominant genus in the phylum *Proteobacteria* was the facultatively anaerobic *Escherichia-Shigella*, whose abundance decreased after the *C. jejuni* challenge. This was in contrast to the genus *Clostridia* UCG-014, whose relative abundance increased after the *C. jejuni* challenge. Similar findings were reported by Awad et al. (2016), where 14-day-old Ross 308 broilers were challenged with 1 × 10^8^ CFU of *C. jejuni* NCTC 12744. In that study, *C. jejuni* colonization was associated with an alteration of the gut microbiota with infected birds having a significantly lower abundance of *Escherichia coli*, while the level of *Clostridium* spp. was higher in infected birds compared to non-infected. However, it should be noted that the higher abundance of *Clostridium spp*. induced after the *C. jejuni* challenge was not straightforward in the present study, since a concomitant decrease in the relative abundance of the genus *Clostridia* vadinBB60 group was observed. Notably, 23 out of the top 25 genera were commonly observed in both R-308 and RR. In spite of this fact, high individual variation in the birds’ cecal microbiota composition within the same broiler type, treatment group, and at the same infection time point in relation to the *C. jejuni* challenge was observed. This is in agreement with a previous study where great individual microbiota variation between animals within a single uniformly derived and treated group, under highly controlled experimental conditions, was reported (Stanley et al., 2014). The authors of that study speculate that the likely reasons for this variation are the lack of exposure to maternally obtained bacteria and the sensitivity to colonization by environmental bacteria in hatcheries.

Our aim with this study was to compare different strategies to administer LAB in order to increase their content in the broilers’ gut and create an unfavorable environment for *Campylobacter*. The relative abundance of genus *Lactobacillus* in both Experiments was relatively high, the twelfth most abundant bacteria in the gut in Experiment 1 and the ninth most abundant in Experiment 2. However, neither the addition of silage LAB (including *Lactiplantibacillus plantarum* 256) nor the *L. plantarum* 256 supplemented in water affected the relative abundance of *Lactobacillus* in broilers’ ceca compared to the control birds that did not receive any LAB. Therefore, we speculate that the initial inhibition of *C. jejuni* growth in feces observed in the silage group in Experiment 1, may be pH dependent rather than due to the presence of LAB. In contrast, a possible effect of *C. jejuni* colonization on the *Lactobacillus* abundance was observed in sequencing data, where the relative abundance of the genus *Lactobacillus* decreased after the *C. jejuni* challenge in Experiment 1, independent of treatment. With the possible impact of lower *C. jejuni* abundance with time after the challenge, the highest presence of *Lactobacillus* was observed at 20 dpi. Interestingly, a linkage between the genera *Lactobacillus* and *Campylobacter* has been previously reported by Sofka et al. (2015), where LAB were found to be significantly higher, in total cultural colony counts, in *Campylobacter-negative* samples from broiler flocks in comparison to the *Campylobacter-positive* ones. Yet, the same correlation was not observed in Experiment 2, where the relative abundance of genus *Lactobacillus* increased after the *C. jejuni* challenge, and its abundance was increasing with time after the infection, independently of treatments. Moreover, even if the genus *Lactobacillus* had at −1 dpi relatively high abundance in RR ceca in the silage and LP256 group in comparison to the control (not significant), no effect of its presence on *Campylobacter* loads was observed after the infection.

Despite the fact that all tested birds were culture negative for *Campylobacter* the day before infection (−1 dpi), we unexpectedly observed a relative abundance of 0.01% of the genus *Campylobacter* in both experiments at −1 dpi. Because the same minor presence of this genus was observed in −1 dpi samples in both experiments, although birds were sampled 1 week apart, and no *C. jejuni* were detected by culture, it is highly unlikely that the 16S amplicon sequence variants reflect the actual presence of *C. jejuni*. If *C. jejuni* would have been present in the stable before the challenge, the level of abundance detected by 16S amplicon sequencing would likely differ between Experiments 1 and 2. Furthermore, the abundance of *C. jejuni* would have been higher since it is well established that after infection, broilers rapidly accumulate high numbers of *C. jejuni* in the cecal content within 3 days (Shanker et al., 1990). Therefore, we assume that contamination in DNA extraction has occurred.

In conclusion, the current study shows that grass silage inoculated with *L. plantarum* 256 (provided as TMR) or water supplemented with *LP*256 are not effective interventions against *C. jejuni* colonization in R-308 or RR broilers. Yet, the minor reductions in *C. jejuni* observed at 1 dpi in fecal samples from R-308 suggest that this approach could still be explored and optimized for better effects. However, it should be noted that due to the cfu/g expression, the reduction in *C. jejuni* load in one group may be artificially biased compared to the other groups.

Further optimization could involve a change from a grass-based silage to a wheat-based silage, as the latter is likely to be more palatable to the birds and hence result in a larger amount of LAB consumed. This could potentially induce greater gut colonization and a stronger inhibitory effect on *C. jejuni*. However, further research is needed to confirm this hypothesis, together with the evaluation of wheat-based silage inclusion levels on the broiler’s performance. It is evident from this work that *C. jejuni* presence as well as broiler type and age had much greater effects on the cecal microbiota composition than the different feed additives.

## Data availability statement

The data presented in the study are deposited in the NCBI repository, accession number PRJNA876811, https://www.ncbi.nlm.nih.gov/bioproject/PRJNA876811.

## Ethics statement

The animal study was reviewed and approved by the Uppsala Ethics Committee for Animal Research, Uppsala, Sweden. Protocol number 5.8.18-16271/2017.

## Author contributions

HWall, EI, KK, and PE obtained the funding and designed the experiments, with HWall as the project leader. EV, HWall, EI, PE, HWang, and FD obtained samples during the study. EV analyzed, interpreted, and compiled the data. LS performed the bioinformatics analysis. FD and HWang performed the plating, DNA extraction, and qPCR analysis. EV wrote the major part of the manuscript, and PE and LS some parts. All authors contributed to the article and approved the submitted version.

## Funding

This work was supported by the SLU EkoForsk, Swedish University of Agricultural Sciences (2016.4.1-742-10).

## Conflict of interest

The authors declare that the research was conducted in the absence of any commercial or financial relationships that could be construed as a potential conflict of interest.

## Publisher’s note

All claims expressed in this article are solely those of the authors and do not necessarily represent those of their affiliated organizations, or those of the publisher, the editors and the reviewers. Any product that may be evaluated in this article, or claim that may be made by its manufacturer, is not guaranteed or endorsed by the publisher.
