# Supplementary material for: Intestinal colonization with Campylobacter jejuni affects broiler gut microbiota composition but is not inhibited by daily intake of Lactiplantibacillus plantarum
Source: Front Microbiol. 2023 Jul 28;14:1205797. doi: 10.3389/fmicb.2023.1205797 (PMC10416237; doi:10.3389/fmicb.2023.1205797)
Supplement: Supplementary file 1 [file Data_Sheet_1.zip › Figure S1.pdf]

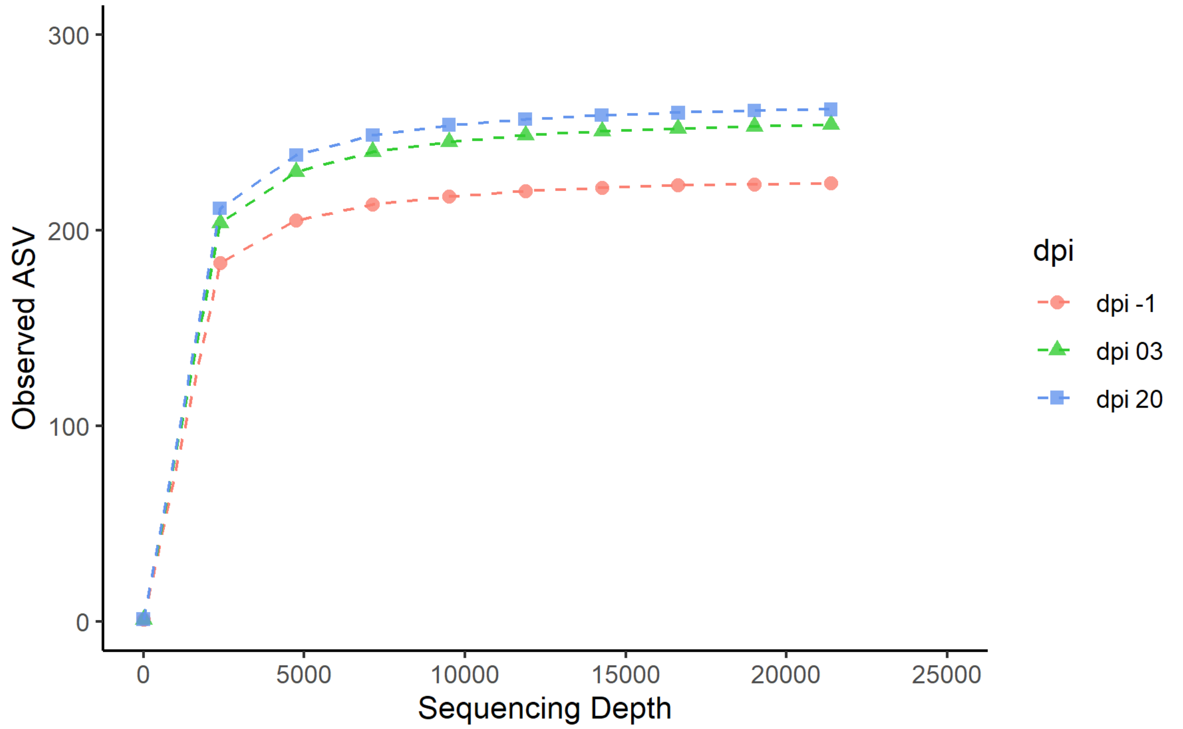

Supplementary Figure 1. The rarefaction curves of observed ASVs in cecal samples in Experiment 1 (Ross 308) at day post-infection (dpi) -1, 3, and 20; represented by 20 cecal samples respectively. Dpi -1 is represented by samples taken before the *C. jejuni* challenge.
