# Supplementary material for: Intestinal colonization with Campylobacter jejuni affects broiler gut microbiota composition but is not inhibited by daily intake of Lactiplantibacillus plantarum
Source: Front Microbiol. 2023 Jul 28;14:1205797. doi: 10.3389/fmicb.2023.1205797 (PMC10416237; doi:10.3389/fmicb.2023.1205797)
Supplement: Supplementary file 1 [file Data_Sheet_1.zip › Table S3.DOCX]

| Supplementary Table 3. Standard curve generated for qPCR | | | | | | | |
| --- | --- | --- | --- | --- | --- | --- | --- |
| Sample | cfu | dilution | CT1 | CT2 | CT3 | CT mean | STDev |
| C. jejuni #65 | 3.30E+07 | 0.00E+00 | 22.31 | 22.37 | 22.44 | 22.37 | 0.061418 |
|  | 3.30E+06 | 1.00E-01 | 25.71 | 25.79 | 25.74 | 25.75 | 0.040553 |
|  | 3.30E+05 | 1.00E-02 | 29.19 | 29.24 | 29.13 | 29.19 | 0.058444 |
|  | 3.30E+04 | 1.00E-03 | 32.25 | 32.26 | 32.21 | 32.24 | 0.025633 |
